# Diminution of 37-kDa laminin binding protein expression reduces tumour formation of murine lung cancer cells

**DOI:** 10.1038/sj.bjc.6690474

**Published:** 1999-06

**Authors:** K Satoh, K Narumi, T Abe, T Sakai, T Kikuchi, M Tanaka, T Shimo-Oka, M Uchida, F Tezuka, M Isemura, T Nukiwa

**Affiliations:** Departments of Respiratory Oncology and Molecular Medicine, and Pathology, Institute of Development, Aging and Cancer, Tohoku University, Aoba-ku, Sendai, 980-8575, Japan; Life Science Center, Iwaki Glass Co. Ltd, Funabashi, 273-0044, Japan; Department of Biochemistry, School of Food and Nutritional Sciences, University of Shizuoka, Shizuoka, 422-0000, Japan

**Keywords:** laminin receptor, 37LBP/p40, mouse, lung cancer, antisense RNA, tumorigenicity

## Abstract

Expression of the 37-kDa laminin binding protein (37LBP/p40), a precursor of the 67-kDa laminin receptor, is well-correlated with the biological aggressiveness of cancer cells. To elucidate the direct role played by 37LBP/p40 in cancer cells, a murine lung cancer cell line T11, the 37LBP/p40 expression of which was remarkably diminished, was established by the introduction of the antisense 37LBP/p40-RNA using a retroviral vector. As a result, the population doubling time of T11 was prolonged (60 h) compared with that of P29, the non-transfected parental cell line (42 h), and TN2, a transfectant with vehicle only (40 h). In-vitro studies also showed that T11 cells adhered to immobilized laminin less firmly than P29 cells did. When 5 × 10^5^ cells were subcutaneously inoculated into syngenic mice, the mean survival time of T11-recipients (77.0 ± 14.8 days) was also significantly prolonged compared with that for P29 (34.8 ± 5.5 days) and TN2 (36.7 ± 6.1 days) recipients (*P* < 0.001). The electron-microscopic view of the tumour tissue revealed that T11 cells were loosely apposed and their intercellular space was markedly widened. Some of the T11 cells sporadically degenerated with the infiltration of lymphocytes and neutrophils. These results suggest that the suppressed expression of 37LBP/p40 reduces the capability of lung cancer cell proliferation in vitro and tumour formation in vivo. © 1999 Cancer Research Campaign


*British Journal of Cancer* (1999) **80**(8), 1115–1122
© 1999 Cancer Research Campaign
Article no. bjoc.1999.0474

### Diminution of 37-kDa laminin binding protein expression reduces tumour formation of murine lung cancer cells

**K Satoh[1], K Narumi[1], T Abe[1], T Sakai[1], T Kikuchi[1], M Tanaka[1], T Shimo-Oka[3], M Uchida[3], F Tezuka[2], M Isemura[4] and T Nukiwa[1]**

Departments of [1]Respiratory Oncology and Molecular Medicine, and [2]Pathology, Institute of Development, Aging and Cancer, Tohoku University, 4-1 Seiryo-Machi, Aoba-ku, Sendai 980-8575, Japan; [3]Life Science Center, Iwaki Glass Co., Ltd, Funabashi 273-0044, Japan; [4]Department of Biochemistry, School of Food and Nutritional Sciences, University of Shizuoka, Shizuoka 422-0000, Japan

**Summary** Expression of the 37-kDa laminin binding protein (37LBP/p40), a precursor of the 67-kDa laminin receptor, is well-correlated with the biological aggressiveness of cancer cells. To elucidate the direct role played by 37LBP/p40 in cancer cells, a murine lung cancer cell line T11, the 37LBP/p40 expression of which was remarkably diminished, was established by the introduction of the antisense 37LBP/p40-RNA using a retroviral vector. As a result, the population doubling time of T11 was prolonged (60 h) compared with that of P29, the non-transfected parental cell line (42 h), and TN2, a transfectant with vehicle only (40 h). In-vitro studies also showed that T11 cells adhered to immobilized laminin less firmly than P29 cells did. When $5 \times 10^5$ cells were subcutaneously inoculated into syngenic mice, the mean survival time of T11-recipients (77.0 ± 14.8 days) was also significantly prolonged compared with that for P29 (34.8 ± 5.5 days) and TN2 (36.7 ± 6.1 days) recipients ($P < 0.001$). The electron-microscopic view of the tumour tissue revealed that T11 cells were loosely apposed and their intercellular space was markedly widened. Some of the T11 cells sporadically degenerated with the infiltration of lymphocytes and neutrophils. These results suggest that the suppressed expression of 37LBP/p40 reduces the capability of lung cancer cell proliferation in vitro and tumour formation in vivo.

**Keywords**: laminin receptor; 37LBP/p40; mouse; lung cancer; antisense RNA; tumorigenicity

It is well-known that the interaction between laminin, a major glycoprotein component of the basement membrane, and laminin receptor, a member of the integrin family, on the cancer cell surface is one of the crucial factors during tumour growth, invasion and metastasis (Hunt, 1989). Besides the integrin family, the laminin binding protein (37LBP/p40), the molecular size of which is 37–40 kDa, has been described. 37LBP/p40 has been proven to be a precursor protein of the non-integrin 67-kDa laminin receptor (67LR) (Wewer et al, 1987; Sobel, 1993) as demonstrated by pulse-chase and transfection experiments (Castronovo et al., 1991*a*, 1991*b*).

Expression of 37LBP/p40 is reported to be up-regulated in human colon cancer (Castronovo et al, 1992; Kim et al, 1998) and breast cancer (Shi et al, 1993; Menard et al, 1994). In previous reports including ours, increased expression of 37LBP/p40 has also been demonstrated in human lung cancer, especially in the highly aggressive cell types such as small-cell and alveolar-cell cancer (Satoh et al, 1992; Ferrarini et al, 1994; Pellegrini et al, 1994).

On the other hand, 37LBP/p40 appears to be a multifunctional protein involved in the translational machinery and identified as p40 ribosome-associated protein (Auth and Brawerman, 1992; Davis et al, 1992; Demianova et al, 1996). Thus, 37LBP/p40 is still controversial, and detailed functions of 37LBP/p40 are not yet fully understood (Mercurio and Shaw, 1991; Yang et al, 1992; Demianova et al, 1996).

Most recently, the gene for 37LBP/p40 has been cloned (Jackets et al, 1996). Interestingly, the active gene is localized on chromosome 3 in the locus 3p21.3, which is a hot spot for genetic alterations in several cancers, particularly in small-cell lung cancer (Karp and Broder, 1995). In this context, it is very alluring to elucidate the role played by 37LBP/p40 in lung cancer.

In the present study, we established a murine lung cancer cell line, with the 37LBP/p40 production being extremely inhibited by the introduction of antisense RNA from reverse-orientated-37LBP/p40 cDNA. As a result, the population doubling time of the transfectant with 37LBP/p40 antisense RNA was prolonged as compared with the parental cells without transfection in vitro. In addition, the primary tumour growth of transplanted cells with antisense 37LBP/p40 RNA was retarded in syngenic mice. These observations suggest a promotive role of 37LBP/p40 in the proliferation and tumorigenicity of lung cancer cells.

### MATERIALS AND METHODS

### Cells and vector

Murine lung cancer cell line P29 (Nakanishi et al, 1992) derived from Lewis lung carcinoma (3LL) was a generous gift of Dr M Okayama (Kyoto, Japan). Retroviral vector N2 and its packaging cell line Ψ2 (Eglitis et al, 1985) were provided by Dr R Mulligan (MIT, Cambridge, MA, USA).

*Received 28 October 1996*
*Revised 5 January 1999*
*Accepted 6 January 1999*

*Correspondence to:* K Satoh

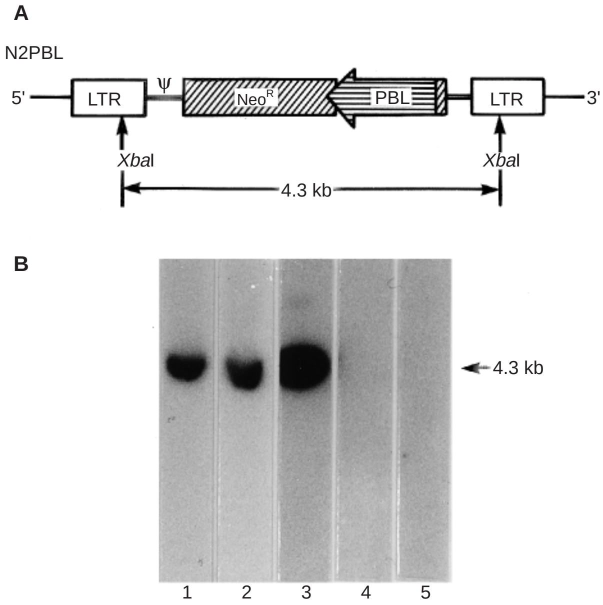

**Figure 1** Construction of vector and transfection into P29 cells. (**A**) Construction of the antisense RNA expression vector N2PBL. Complementary DNA for 37LBP/p40 was obtained from P29 cells by the method of RT-PCR and was subcloned into the *Xho*I site of retroviral vector N2 (Eglitis et al., 1985) in opposite orientation as designated PBL in contrast to 37LBP/p40. Sense RNA of the neomycin-resistant gene and antisense RNA of the 37LBP/p40 gene were expressed under the direction of the 5′-LTR promoter. ψ indicates a packaging signal. (**B**) Southern hybridization of the genomic DNA digested with *Xba*I. There is no *Xba*I site in the plasmid construction except for two in the LTR sequence. After transfer of digested genomic DNA, the nylon membrane filter was hybridized with radiolabelled 37LBP/p40 cDNA probe. The expected 4.3-kb bands containing genes of neomycin-resistance and 37LBP/p40 cDNA were shown in T11 (lane 1) and T15 (lane 2) cells. Note that no positive band was found in TN2 (transfectant with N2 vector only; lane 4) and in P29 (parental cell without transfection; lane 5). The plasmid pN2PBL was shown as a positive control (lane 3)

### DNA and plasmid construction

Full-length murine 37LBP/p40 cDNA (pKN57) was obtained by reverse transcription polymerase chain reaction (RT-PCR) (Robinson and Simon, 1991) of the total RNA of the P29 cells. Two oligonucleotide primers for RT-PCR (LNR-14: 5´ATAGCTCGAG-CTTGATTCCCATCGTAACTT3´; LNR-15: 5´ACCACTCGAGTGG-TGGCTCCAACCCACTCA3´) were synthesized according to the sequence of murine 37LBP/p40 cDNA (Rao et al, 1989) with the *Xho*I site at each 5´ end. The nucleotide sequence of the cDNA insert was confirmed by the dideoxy chain termination method. The retroviral vector used for the introduction of the antisense RNA was constructed from N2 as shown in Figure 1A. An insert of the pKN57 (940-bp) was subcloned in reverse direction in the *Xho*I site of N2 by a standard technique and was designated pN2PBL.

### Transfection and infection

*Psi*2 cells were grown in non-selective Eagle's modified essential medium (EMEM) containing 2 mM glutamine and 10% calf serum at 37°C under 10% carbon dioxide. Plasmid pN2PBL was transfected into Ψ2 cells by the calcium phosphate method (Miller et al, 1985). The cells were grown in selective medium containing G418 (neomycin sulphate) at a concentration of 0.3 g $l^{-1}$ to select neomycin-resistant clones. The selected clones (Ψ 2PBL) packaged the transcripts from the integrated pN2PBL into ecotropic retroviral particles. P29 cells were infected by the culture supernate of the Ψ 2PBL cells (titre > $10^5$ cfu $ml^{-1}$) as described by Miller et al. (1985) and cultured in selective medium (EMEM, 2 mM glutamine, 10% fetal calf serum with 0.5 g $l^{-1}$ G418) at 37°C under 5% carbon dioxide. Fifty neomycin-resistant P29 clones, referred to as Transfectant 1–50 (T1–T50), were isolated using a cloning ring and were used for the following experiments. Some P29 cells were infected with N2 vehicle only and cloned as a negative control polyclonal cell line referred to as TN2. For the determination of the population doubling time, cells were plated in a 35 mm-diameter dish at an initial density of $10^3$ cells. The absence of mycoplasma infection in each cell line was confirmed by a Gene-Probe mycoplasma detection kit (Chugai, Tokyo, Japan).

### DNA and RNA analysis

To confirm the integration of pN2PBL into the genome of transfectant cells, Southern analysis was carried out using the genomic DNA of the transfectant cells digested with restriction enzyme *Xba*I, for which the recognition site was located within both LTR sequences. The blotted nylon membrane Nytran (Schleicher and Schuell, Keene, NH, USA) was hybridized with a radiolabelled 37LBP/p40 cDNA probe (pKN57). Endogenous gene expression of 37LBP/p40 was evaluated using total RNA of the cells by Northern blot analysis with the cDNA probe described above.

### Immunostaining

Cells ($1 \times 10^5$) were seeded onto a LAB-TEK chamber (Nunc, Roskilde, Denmark) and cultured for 24 h. After washing and fixing with cold acetone, the LAB-TEK chamber was immunostained with a polyclonal antibody P1 that was raised against the C-terminal end of the human 37LBP/p40 polypeptide (PTED-WSAQPATEDWSAAPTA in one-letter amino acid symbols), which has been described previously (Rao et al, 1989).

### Western blot analysis

Total cellular proteins were extracted by a method using a solution containing detergents and various kinds of protease inhibitor. The cell lysates were clarified by centrifugation and electrophoresed on 7.5% sodium dodecyl sulphate (SDS) polyacrylamide gel (Laemmli, 1970), followed by immunoelectroblotting onto a Nytran. Immunoblotting was carried out with the polyclonal antibody described above. For normalization of 37LBP/p40 protein expression, the same filter was blotted with anti-β-actin antibody (Santa Cruz Biotechnology, CA, USA).

### FACScan analysis

Cells were harvested and analysed by flow cytometry according to the method of Landowski et al (1995). Briefly, unpermeabilized cells were incubated with the murine monoclonal anti-human 37LBP/p40 antibody M1 (Iwaki-Grass, Funabashi, Japan), which was converted from a polyclonal antibody P1, or with PBS for 60 min on ice. After pelleting the cells and resuspending them in PBS, the secondary antibody, fluorescein isothiocyanate

 © 1999 Cancer Research Campaign

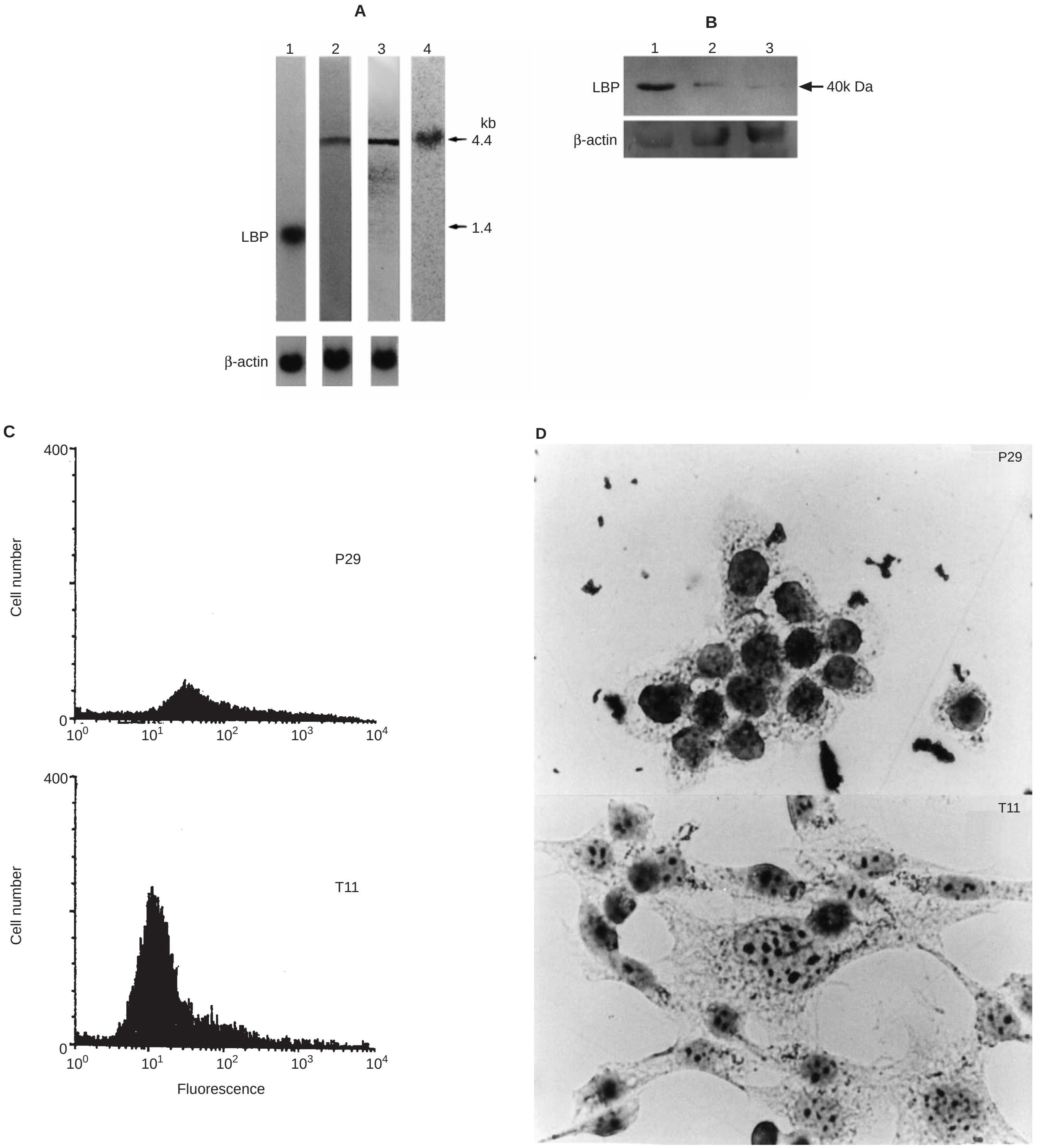

**Figure 2** Inhibition of endogenous 37LBP/p40 expression in transfectant T11 by antisense RNA. (**A**) Northern blot analysis of 37LBP/p40. Fifteen micrograms of total RNA per lane was electrophoresed and transferred onto nylon membrane. Hybridization was performed with radiolabelled 37LBP/p40 cDNA probe. Note that endogenous gene product (1.4 kb) was remarkably reduced in T11 (lane 2) and T15 (lane 3) cells compared with controls P29 (lane 1). Bands (4.4-kb) seen in both lane 2 and lane 3 were regarded as exogenous antisense transcripts for 37LBP/p40. An antisense 37LBP/p40 transcript of T11 was also indicated by Northern hybridization with sense oligonucleotide probe (lane 4). The same membrane was hybridized with β-actin probe as a control (lanes 1–3). (**B**) Western blot analysis of 37LBP/p40. Whole extract of $1 \times 10^5$ cells was electrophoresed on 7.5% SDS-polyacrylamide gel. Note that the synthesis of the 37LBP/p40 was suppressed in both T11 and T15 cells (lanes 2 and 3 respectively) compared with P29 (lane 1). The same membrane was immunoblotted with anti-β-actin antibody for normalization of 37LBP/p40 protein expression. (**C**) FACScan analysis of the surface expression of the 37LBP/p40 protein. Cells were treated with anti-37LBP/p40 antibody and stained with FITC-conjugated goat anti-murine secondary antibody. Endogenous expression of 37LBP/p40 was decreased on the cell surface of antisense-transfectant T11 (lower panel) compared with that of parental cell P29 (upper panel). Ordinate and abscissa indicate the cell number and the fluorescence intensity respectively. (**D**) Difference in morphology between P29 and T11 cells. Cells were photographed 12 h after plating in plastic culture dish. T11 cells (lower panel) appear larger than parental P29 cells with rich cytoplasm (upper panel). Original magnification is × 100

© 1999 Cancer Research Campaign

(FITC)-labelled goat anti-murine immunoglobulin (Sigma), was added and allowed to react for 60 min on ice. Analysis was performed on a Becton Dickinson FACScan (Franklin Lakes, NJ, USA). The transfected cells were assayed as soon as the population had stabilized after G418 selection.

### Assays for attachment and detachment to immobilized laminin

Cell adherence to laminin was evaluated by the method of Nakanishi et al (1992). In attachment assay, cells ($2.5 \times 10^5$ per well) were plated onto a 24-well laminin-coating dish (Gibco BRL, Rockville, MD, USA) and allowed to adhere. At various time points, unattached cells were carefully removed by washings with PBS, and the remaining adherent cells were recovered by 0.01% (w/v) trypsin to be counted. Cell attachment was shown as the ratio of remaining cell count/initial cell count ($2.5 \times 10^5$). In detachment assay, cells ($2.5 \times 10^5$ per well) were plated onto a laminin-coating dish and incubated for 20 h. The cell layer was washed twice with PBS and then rotated on an orbital shaker (Marisol, Tokyo, Japan) in PBS containing 0.002% (w/v) trypsin at 100 rpm for the indicated times. The percentage of detached cells was calculated with the cell count at 0 min.

### Inoculation of the cells

Cells ($5 \times 10^5$) were subcutaneously injected into the left shoulders of syngenic mice C57BL/6 (ten animals per group). Growth of the primary tumour was monitored every 5 days by caliper measurement, and mice were observed until they died from tumour development. To indicate the expression of the 37LBP/p40 protein in the transplanted tumour, a Western blot of the T11 tumour was also carried out. Metastases to the lung, liver and axillar lymph node were macroscopically observed in T11 and P29 recipients (five mice per group) on day 35.

### Electron-microscopical analysis

Primary subcutaneous tumour tissues were fixed in 2.5% glutaraldehyde, 2.0% paraformaldehyde, 0.1 M phosphate buffer (pH 7.3) for 2 h, post-fixed in 1% osmium tetroxide, and embedded in Epon 812 (Taab, Berkshire, UK). Ultra-thin sections were stained with uranyl acetate followed by lead citrate and examined by an electron microscope (Hitachi, H-7100).

### Statistical analyses

The significance of differences in the mean survival time of mice transplanted with three cell lines was assessed with ANOVA.

### RESULTS

### Construct of antisense 37LBP/p40 RNA transfectant

Fifty neomycin-resistant clones were obtained by G418 selection. Two of them (T11 and T15) transcribed antisense RNA from the pN2PBL transferred by retroviral vector. Southern blot analysis indicated that the pN2PBL insert was integrated into the genomes of T11 and T15 cells (Figure 1B). No insert DNA was demonstrated in the genomes of P29 and TN2 cells. Integration of pN2PBL was also substantiated by RT-PCR analysis using a specific primer for the NeoR gene (Beck et al, 1982) and LNR-14 primer described above. Northern and Western blot analyses showed that endogenous expressions of 37LBP/p40 were remarkably suppressed in both antisense RNA-transfectant T11 and T15 (Figure 2 A,B). We thereafter chose T11 for experiments because suppression of the endogenous 37LBP/p40 expression was greater in T11 than in T15.

### Phenotype of antisense RNA transfectant T11

To estimate the expression of 37LBP/p40 protein on the cell surface, a FACScan analysis was performed using parental cells P29 and antisense-transfectant T11 (Figure 2C). As a result, the expression of the 37LBP/p40 protein of T11 decreased compared with that of P29, supporting the results of the Northern and Western analyses. The immunoreactivity of the T11 cells was far lower than that of the P29 cells, consistent with the Western blot analysis of whole cell extract as shown in Figure 2B (data not shown). Morphologic characteristics of the cell lines are shown in Figure 2D. Parental P29 cells were relatively small and round (upper panel), while T11 cells were larger than the P29 cells with rich cytoplasm (lower panel).

T11 showed a prolonged doubling time. From the logarithmic phase of the cell growth curve, the population doubling times of P29 (parental cells), TN2 (vehicle-transfectant), and T11 were calculated to be 42 h, 40 h, and 60 h respectively. This suggests that the suppressed expression of 37LBP/p40 reduced the proliferation rate of the T11 cells. The differences in doubling time were also documented even in the medium containing an excess amount of EHS laminin (10 ng ml$^{-1}$), indicating that the proliferation rate of each cell line was not significantly changed by exogenous laminin.

### Cell adherence to immobilized laminin

In vitro analyses showed that the initial rate and extent of attachment of T11 cells to immobilized laminin were lower than those of P29 cells (Figure 3A). To demonstrate the difference in the potential for laminin attachment, resistance to detachment was also estimated by trypsin-treatment. As shown in Figure 3B, T11 cells (■) were less resistant to trypsinization than P29 cells (•) in the laminin-coating dish.

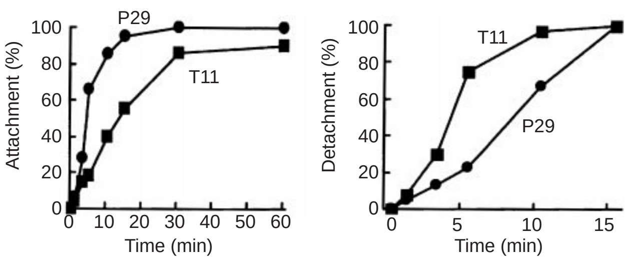

**Figure 3** Cell attachment and detachment to immobilized laminin. Results are presented as the mean value of triplicate. Standard deviations were less than 10% of the mean. (**A**) Attachment assay. Note that both the initial rate and the extent of attachment of T11 (■) were lower than those of P29 (●). (**B**) Detachment assay by trypsin treatment. T11 (■) was less resistant to trypsinization than P29 (●) on the laminin-coating dish

© 1999 Cancer Research Campaign

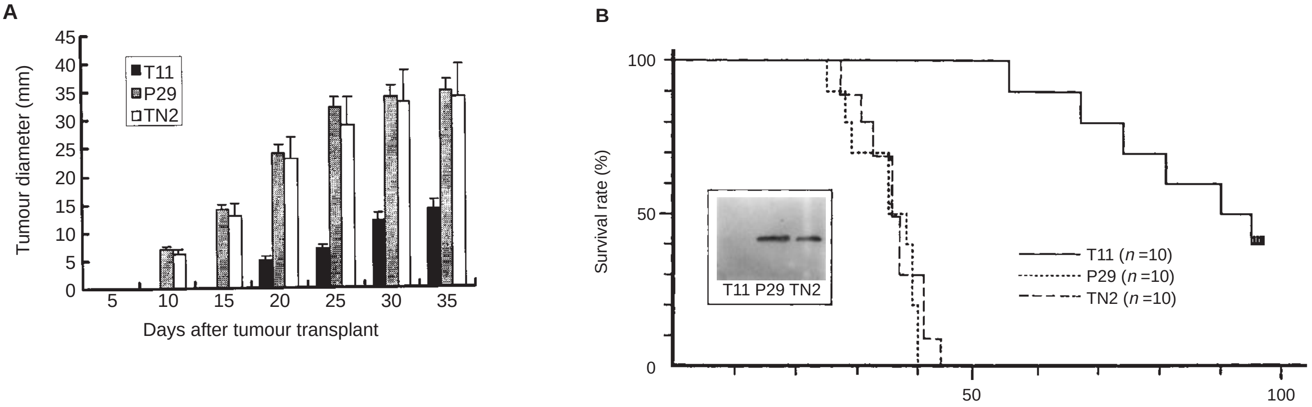

**Figure 4** In-vivo effect of antisense 37LBP/p40 RNA in syngenic mice. Cells ($5 \times 10^5$) were subcutaneously injected into the left shoulder of a male C57BL/6 syngenic mouse. (**A**) Take of tumour in recipient mouse was estimated in regard to the tumour diameter. The T11 tumour (closed bar) grew more slowly than both the P29 (dotted bar) and TN2 (open bar) tumours. (**B**) The survival curve (Kaplan–Meier's plot) was obtained using ten mice per group. The mean survival time of P29, TN2, and T11 were calculated at 34.8 ± 5.5 days, 36.7 ± 6.1 days, and 77.0 ± 14.8 days respectively. The tumorigenicity with regard to the mortality of T11 was significantly reduced compared with those of P29 and TN2 ($P < 0.001$). Inset: Western blot of T11 transplanted tumour indicates an extremely reduced expression of the 37-kDa protein as well as that of T11 cells

### Subcutaneous inoculation into syngenic mice

The tumorigenicity of each cell line in vivo was investigated with respect to the mortality due to tumour growth in recipient mice. Inoculated T11 tumour grew more slowly than P29 and TN2 tumours with respect to tumour diameter (Figure 4A). There was a remarkable difference in the survival curves between syngenic mice inoculated with T11 cells and those inoculated with P29 cells and TN2 cells (Figure 4B). Ten recipients of P29 and TN2 were all dead within 40 days, indicating no significant difference in tumorigenicity between these two groups. In contrast, tumour growth in recipients of T11 was far slower, and some mice survived over 120 days. The expression of the 37-kDa protein was extremely reduced in T11 tumours generated in syngenic mice as well as in T11 cells (Figure 4B, inset). When animals (five mice each) were sacrificed 35 days after the subcutaneous inoculation of the cells, macroscopic metastases were observed in the lung, liver and the axillar lymph nodes of P29-recipients. In contrast, no metastasis was seen in T11-recipients (Table 1).

### Histological analysis of tumour tissue

Electron-microscopically, P29 formed groups of epithelial neoplastic cells (Figure 5A). They were polygonal and closely apposed and had small intercellular junctions. They had a relatively small amount of cytoplasm, and the main components of the cytoplasm were free ribosomes, mitochondria and rough endoplasmic reticulum. Nuclei were large, irregularly shaped, and had large nucleoli. Electron-microscopical findings for TN2 cells infected with vehicle only were generally similar to those for P29 cells (data not shown). T11 cells were, in contrast, loosely apposed, and their intercellular space was markedly widened (Figure 5B). The intercellular junctions were few, and the development of microvilli was prominent. Some of the T11 cells sporadically degenerated, and the infiltration of lymphocytes and neutrophils was observed (Figure 5C).

### DISCUSSION

The aim of the present study was to try to diminish the expression of 37LBP/p40 protein expression in cancer cells in order to better understand its role in tumorigenicity. As a result, we established a cell line T11 derived from murine Lewis lung carcinoma with a depletion of 37LBP/p40 by antisense RNA, and demonstrated a phenotype of reduced tumorigenicity. The clone T11 differs from the parental cell line P29 in the following points. First, T11 cells are less closely apposed, their intercellular spaces are markedly widened, and their cell borders are irregular with a prominent development of microvilli, while P29 cells are polygonal and tightly apposed with numerous small intercellular junctions. Secondly, the cell proliferation rate of T11 is lower than that of P29 in vitro. Finally, the tumorigenicity of T11 is far diminished compared to that of P29 when inoculated into syngenic mice.

The relationship between the reduced 37LBP/p40 expression and the morphological and tumorigenic changes of T11 cells has not yet been fully elucidated, but we suggest it to be as follows.

**Table 1** Autopsy of recipient mice on day 35 after tumour inoculation

| Tumour cell | Body weight (g) | Tumour | | Metastasis | | |
|---|---|---|---|---|---|---|
| | | Diameter[a] (cm) | Weight[a] (g) | Lung[a] | Liver[a] | Lymph node |
| P29 (*n* = 5) | 48.40 ± 1.67 | 3.44 ± 0.44 | 14.54 ± 4.85 | 2.80 ± 1.92 | 2.40 ± 1.14 | 0.20 ± 0.45 |
| T11 (*n* = 5) | 43.88 ± 4.94 | 1.0 ± 0.7 | 0.97 ± 0.72 | 0 | 0 | 0 |

[a]$P < 0.001$

© 1999 Cancer Research Campaign 

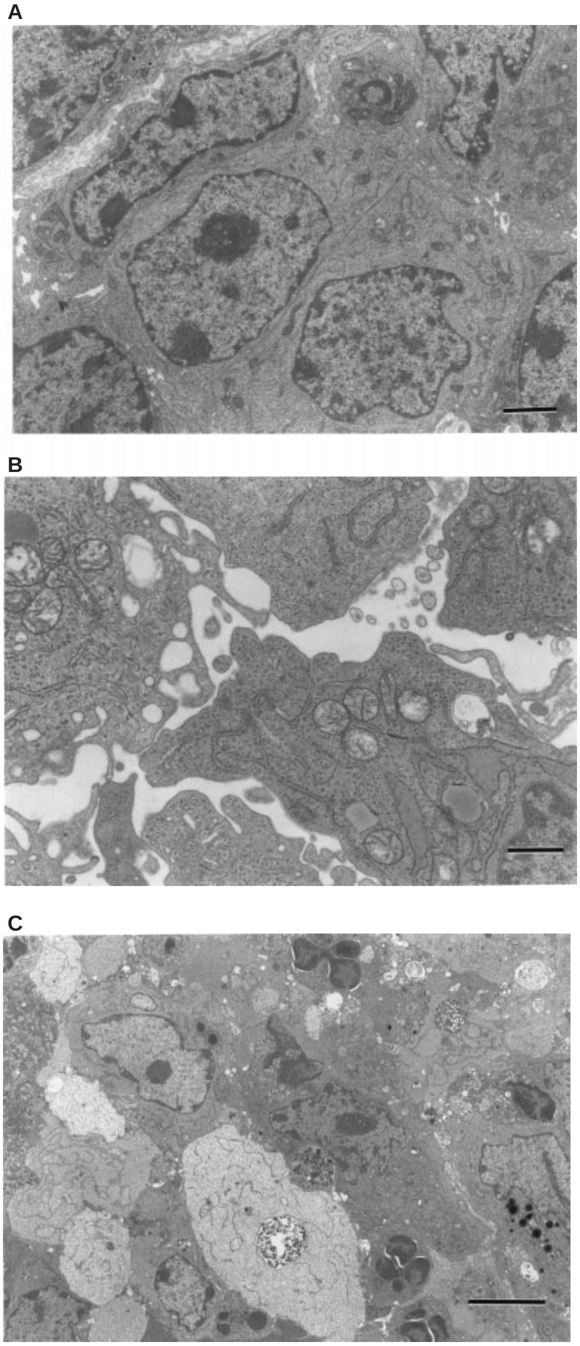

**Figure 5** Electron micrographs of the primary tumour of P29 and T11 cells. (**A**) P29 cells are polygonal, tightly apposed, and have small intercellular junctions (× 4000). The bar indicates 20 μm. (**B**) T11 cells show markedly widened intercellular spaces and a prominent development of microvilli (× 10 000). The bar indicates 40 μm. (**C**) Degenerated cells appear sporadically in the T11 tumour with infiltrations of lymphocytes and neutrophils (× 2000). The bar indicates 10 μm

1. The formation of the tumour matrix and/or complete cell–cell contact might be affected due to defective laminin binding ability of the T11 cells. A loose structure formation of the extracellular matrix was observed in our preliminary study in T11 tumour tissue by immunohistochemistry using anti-laminin and anti-collagen IV antibodies. In addition, a widened intercellular space and a poorly developing desmosome were demonstrated by electron microscopy, as shown in Figure 5.
2. Suppressed angiogenicity of the T11 tumour might be a likely cause of the diminished tumorigenicity. Vacca et al (1993) have indicated that a more frequent interaction between melanoma cells and their microvasculature via laminin is absolutely necessary during tumour progression. Gasparini et al (1995) have also reported a relationship between the expression of 67LR and the density of the intratumoural microvessels, and have shown that measurement of 67LR expression might provide useful prognostic information in human breast cancer.
3. Apoptosis might be induced in the T11 cells by means of the introduction of 37LBP/p40 antisense RNA. The amino acid sequences deduced from 37LBP/p40-cDNA of several species show a dramatically high degree of conservation during evolution (Mercurio and Shaw, 1991). This fact reinforces the idea that the 37LBP/p40 protein might play a fundamental role in cell metabolism. As a consequence, it can be postulated that the 37LBP/p40 protein might play a double function in cells: one as a monomer in the translation machinery and, when modified or associated with another protein, as a laminin binding protein, a receptor for the Sindobis virus (Wang et al, 1992), a receptor for the prion protein (Rieger et al, 1997), or a positional marker protein in the embryonic retina (McCaffery et al, 1990) (N Clausse and V Castronovo, personal communication). Considering these possibilities, it might well be that the diminished level of 37LBP/p40 protein in cells might result in an increase in cell death because the 37LBP/p40 protein would not be present anymore to play its potentially fundamental role as a ribosomal associated protein in an antisense-transfectant T11. In order to assess this possibility, the apoptosis level of the T11 clone in culture should be measured.
4. A reduction in 37LBP/p40 expression might be involved in tumour suppression by the host immune system. A large number of mononuclear cells (lymphocytes and monocytes) infiltrated into the T11 tumour tissue in contrast to P29 and TN2 tumour tissues that contained few mononuclear cells. It is postulated that reduced angiogenesis in the T11 tumour causes tumour autolysis, apoptosis and the resulting accumulation of mononuclear cells in the tissue. On the other hand, strong immune response to DNA by recognition of unmethylated CpG motifs within exogenous oligonucleotide has recently been pointed out (Krieg et al, 1989, 1995). Whether antisense RNA generated from integrated DNA in genome of mammalian cells also triggers host immune system is not clear.
5. It should also be considered that an intracellular marker gene, e.g. *Neo*R, may negatively influence the survival of transplanted cells in vivo (Tapscott et al, 1994). However, TN2 that had been infected only by vehicle with selective marker indicated no significant alterations in phenotype compared with P29.

Antisense strategies have been used previously to diminish or abolish the expression of extracellular matrix receptor other than 37LBP/p40. For example, antisense RNA of $\alpha_2\beta_1$, a collagen/laminin receptor, interferes the organization of breast cancer cells in three-dimensional collagen gels (Keely et al, 1995), and antisense oligonucleotides of $\alpha_1\beta_1$, another collagen/laminin

 © 1999 Cancer Research Campaign receptor, prevents fibroblasts from matrix remodelling seen during morphogenesis and wound healing (Carver et al, 1995).

Besides antisense strategies, the results using peptides to ECM receptors have been reported. It has been known that peptides containing the $\beta_1$ integrin recognition sequence, RGD, can inhibit experimental or spontaneous metastasis (Saiki et al, 1989; Pasqualini et al, 1997). As well as integrin, 37LBP/p40 plays a role in the attachment and migration of tumour cells in vitro to laminin-coated surfaces and in vivo during lung colonization by intravenously injected tumour cells (Wewer et al, 1987). Thus 37LBP/p40 has potentially provided the basis for new anticancer therapeutic strategies. Iwamoto (1987) demonstrated that synthetic YIGSR peptide derived from the sequence of the laminin B1 chain and reported to be a binding site for 37LBP/p40 (Graf et al, 1987), inhibited experimental metastasis of B16 melanoma. Recently, it has been shown that a multimeric form of YIGSR sequence reduced angiogenesis, tumour growth and experimental metastasis of HT1080 more effectively than a monomeric form did (Iwamoto et al, 1996). On the other hand, Castronovo et al (1991*c*) have demonstrated that 37LBP/p40 on the cell surface was reduced by synthetic peptide G, a high-affinity laminin-binding site in the 37LBP/p40, and that adhesion of melanoma cells to the vascular endothelium was inhibited specifically and drastically. They also indicated that a peptide G sequence was necessary to stabilize the laminin binding of cancer cells (Magnifico et al, 1996).

The present study demonstrates that the increase in the survival of T11-inoculated mice is caused cooperatively by (a) delayed time in tumour appearance, (b) reduced growth rate of tumours and (c) no metastasis formation. These results suggest that the suppression of 37LBP/p40 expression reduces tumour formation of lung cancer cells in vivo. However, antisense strategies could affect targets that might be relevant for tumour formation other than 37LBP/p40. Moreover, it cannot be excluded that the observed reduced tumorigenicity might be dependent on some event different from down-regulation of the 37LBP/p40 transcript because the effects generated in this study was assessed by use of a single clone. Therefore, the mechanism of reduced tumorigenicity in association with down-regulation of 37LBP/p40 expression is currently being explored using different cancer cells in addition to T11.

### ACKNOWLEDGEMENTS

We thank Dr R Mulligan for providing N2 and ψ2 cells, and Dr M Okayama for providing P29 cells. We would also like to thank Dr N Clausse and Dr V Castronovo for their helpful remarks. This study was supported by a Grant-in-Aid from the Ministry of Education, Science, and Culture of Japan (#05454250 and #08457178).

### REFERENCES

Auth D and Brawerman G (1992) A 33-kDa polypeptide with homology to the laminin receptor: component of translation machinery. *Proc Natl Acad Sci USA* **89**: 4368–4372

Beck E, Ludwig G, Auerswald EA, Reiss B and Challer H (1982) Nucleotide sequence and exact localization of the neomycin phosphotransferase gene from transposon Tn5. *Gene* **19**: 327–336

Carver W, Molano I, Reaves TA, Borg TK and Terracio L (1995) Role of the alpha 1 beta 1 integrin complex in collagen gel contraction in vitro by fibroblasts. *J Cell Physiol* **165**: 425–437

Castronovo V, Taraboletti G and Sobel ME (1991*a*) Functional domains of the 67-kDa laminin receptor precursor. *J Biol Chem* **266**: 20440–20446

Castronovo V, Claysmith AP, Barker KT, Cioce V, Krutzsch HC and Sobel ME (1991*b*) Biosynthesis of the 67 kDa high affinity laminin receptor. *Biochem Biophys Res Commun* **177**: 177–183

Castronovo V, Taraboletti G and Sobel ME (1991*c*) Laminin receptor complementary DNA-deduced synthetic peptide inhibits cancer cell attachment to endothelium. *Cancer Res* **51**: 5672–5678

Castronovo V, Campo E, vanden Brule FA, Claysmith AP, Cioce V, Liu FT, Fernandez PL and Sobel ME (1992) Inverse modulation of steady-state messenger RNA levels of two non-integrin laminin binding proteins in human colon carcinoma. *J Natl Cancer Inst* **84**: 1161–1169

Davis SC, Tzagoloff A and Ellis SR (1992) Characterization of a yeast mitochondrial ribosomal protein structurally related to the mammalian 68-kDa high affinity laminin receptor. *J Biol Chem* **267**: 5508–5514

Demianova M, Formosa TG and Ellis SR (1996) Yeast proteins related to the p40/laminin receptor precursor are essential components of the 40S ribosomal subunit. *J Biol Chem* **281**: 11383–11391

Eglitis MA, Kantoff P, Gilboa E and Anderson WF (1985) Gene expression in mice after high efficiency retroviral-mediated gene transfer. *Science* **230**: 1395–1398

Ferrarini M, Pupa SM, Zocchi MR, Rugarli C and Menard S (1994) Distinct pattern of HSP72 and monomeric laminin receptor expression in human lung cancers infiltrated by gamma/delta T lymphocytes. *Int J Cancer* **57**: 486–490

Gasparini G, Barbareschi M, Boracchi P, Bevilazqua P, Verderio P, Dalla Palma P and Menard S (1995) 67-kDa laminin-receptor expression adds prognostic information to intra-tumoral microvessel density in node-negative breast cancer. *Int J Cancer* **60**: 604–610

Graf J, Ogle RC, Robey FA, Sasaki M, Martin GR, Yamada Y and Kleinman HK (1987) A pentapeptide from the laminin B1 chain mediates cell adhesion and binds the 67 000 laminin receptor. *Biochemistry* **26**: 6896–6900

Hunt G (1989) The role of laminin in cancer invasion and metastasis. *Exp Cell Biol* **57**: 165–176

Iwamoto Y, Robey FA, Graf J, Sasaki M, Kleinman HK, Yamada Y and Martin GR (1987) YIGSR, a synthetic laminin pentapeptide, inhibits experimental metastasis formation. *Science* **238**: 1132–1134

Iwamono Y, Nomizu M, Ymamada Y, Ito Y, Tanaka K and Sugioka Y (1996) Inhibition of angiogenesis, tumour growth and experimental metastasis of human fibrosarcoma cells HT1080 by a multimeric form of the laminin sequence Tyr–Ile– Gly–Ser–Arg (YIGSR). *Br J Cancer* **73**: 589–595

Jackets P, Minoletti J, Belotti K, Clausse N, Sozzi G, Sobel ME and Castronovo V (1996) Isolation from a multigene family of the active human gene of the metastasis-associated multifunctional protein 37LPR/p40 at chromosome 3p21.3. *Oncogene* **13**: 495–503

Karp JE and Broder S (1995) Molecular foundations of cancer: new targets for intervention. *Nat Med* **1**: 309–320

Keely PJ, Fong AM, Zutter MM and Santoro SA (1995) Alteration of collagen-dependent adhesion, motility, and morphogenesis by the expression of antisense alpha2 integrin mRNA in mammary cells. *J Cell Sci* **108**: 595–607

Kim W, Lee BL, Jun SH, Song SY and Kleinman HK (1998) Expression of 32-67-kDa laminin receptor in laminin adhesion-selected human colon cancer cell lines. *Br J Cancer* **77**: 15–20

Krieg AM, Gause WC, Gourley MF and Steinberg AD (1989) A role for endogenous retroviral sequences in the regulation of lymphocyte activation. *J Immunol* **143**: 2448–2451

Krieg AM, Yi AK, Matson S, Waldschmidt TJ, Bishop GA, Teasdale R, Koretzky GA and Klinman DM (1995) CpG motifs in bacterial DNA trigger direct B-cell activation. *Nature* **54**: 446–549

Laemmli UK (1970) Cleavage of structural proteins during the assembly of the head of bacteriophage T4. *Nature* **227**: 680–685

Landowski TH, Uthayakumar S and Starkey JR (1995) Control pathways of the 67 kDa laminin binding protein: surface expression and activity of a new ligand binding domain. *Clin Exp Metastasis* **13**: 357–372

Magnifico A, Tagliabue E, Buto S, Ardini E, Castronovo V, Colnaghi MI and Menard S (1996) Peptide G, containing the binding site of the 67-kDa laminin receptor, increases and stabilized laminin binding to cancer cells. *J Biol Chem* **271**: 31179–31184

McCaffery P, Neve RL and Draeger UC (1990) A dorso-ventral asymmetry in the embryonic retina defined by protein conformation. *Proc Natl Acad Sci USA* **87**: 8570–8574

Menard S, Squicciarini P, Luini A, Sacchini V, Rovini D, Tagliabue E, Veronesi P, Salvadori B, Veronesi U and Colnaghi MI (1994) Immunodetection of bone marrow micrometastases in breast carcinoma patients and its correlation with primary tumour prognostic features. *Br J Cancer* **69**: 1126–1129

© 1999 Cancer Research Campaign 

Mercurio AM and Shaw LM (1991) Laminin binding protein. *BioEssays* **13**: 469–473
Miller AD, Law MF and Verma IM (1985) Generation of helper-free amphotropic retroviruses that transduce a dominant-acting, methotrexate-resistant dihydrofolate reductase gene. *Mol Cell Biol* **5**: 431–437
Nakanishi H, Takenaga K, Oguri K, Yoshida A and Okayama M (1992) Morphological characteristics of tumours formed by Lewis lung carcinoma-derived cloned cell lines with different metastatic potentials: structural differences in their basement membranes formed *in vivo*. *Virchows Archiv A* **420**: 163–170
Pasqualini R, Koivunen E and Ruoslahti E (1997) αv integrins as receptors for tumor targeting by circulating ligands. *Nature Biotechol* **15**: 542–546
Pellegrini R, Martignone S, Menard S and Colnaghi MI (1994) Laminin receptor expression and function in small-cell lung carcinoma. *Int J Cancer* (suppl), **8**: 116–120
Rao CN, Castronovo V, Schmitt MC, Wewer UM, Claysmith AP, Liotta LA, and Sobel ME (1989) Evidence for a precursor of the high-affinity metastasis-associated murine laminin receptor. *Biochemistry* **28**: 7476–7486
Rieger R, Edenhofer F, Lasmezas CI and Weiss S (1997) The human 37-kDa laminin receptor precursor interacts with the prion protein in eukaryotic cells. *Nat Med* **3**: 1383–1388
Robinson MO and Simon MI (1991) Determining transcript number using the polymerase chain reaction: Pgk-2, mP2, and PGK-2 transgene mRNA levels during spermatogenesis. *Nucleic Acids Res* **19**: 1557–1562
Saiki I, Murata J, Ikda J, Sakurai T, Nishi N, Matsuno K and Azuma I (1989) Antimetastatic effects of synthetic polypeptides containing repeated structures of the cell adhesive Arg–Gly–Asp (RGD) and Tyr–Ile–Gly–Ser–Arg (YIGSR) sequences. *Br J Cancer* **60**: 722–728
Satoh K, Narumi K, Isemura M, Sakai T, Abe T, Matsushima K, Okuda K and Motomiya M (1992) Increased expression of the 67 kDa-laminin receptor gene in human small cell lung cancer. *Biochem Biophys Res Commun* **182**: 746–752
Shi YE, Torri J, Yieh L, Sobel ME, Yamada Y, Lippman ME, Dickson RB and Thompson EW (1993) Expression of 67 kDa laminin receptor in human breast cancer cells: regulation by progestins. *Clin Exp Metastasis* **11**: 251–261
Sobel ME (1993) Differential expression of the 67-kDa laminin receptor in cancer. *Semin Cancer Biol* **4**: 311–317
Tapscott SJ, Miller AD, Olson JM, Berger MS, Groudine M and Spence AM (1994) Gene therapy of rat 9L gliosarcoma; tumours by transduction with selectable genes does not require drug selection. *Proc Natl Acad Sci USA* **91**: 8185–8189
Vacca A, Ribatti D, Roncali L, Lospalluti M, Seri G, Carrel S and Dammacco F (1993) Melanocyte tumor progression is associated with changes in angiogenesis and expression of the 67-kilodalton laminin receptor. *Cancer* **72**: 455–461.
Wang KS, Kuhn RJ, Strauss EG, Ou S, Strauss JH (1992) High-affinity laminin receptor is a receptor for Sindobis virus in mammalian cells. *J Virol* **66**: 4992–5001.
Wewer UM, Taraboletti G, Sobel ME, Albrechtsen R and Liotta LA (1987) Role of laminin receptor in tumor cell migration. *Cancer Res* **47**: 5691–5698.
Yang G, Douville P, Gee S and Carbonetto S (1992) Nonintegrin laminin receptors in the nervous system: evidence for lack of a relationship to p40. *J Neurobiol* **23**: 491–506

*© 1999 Cancer Research Campaign*
